# Systemic modified messenger RNA for replacement therapy in alpha 1-antitrypsin deficiency

**DOI:** 10.1038/s41598-020-64017-0

**Published:** 2020-04-27

**Authors:** Ahmad Karadagi, Alex G. Cavedon, Helen Zemack, Greg Nowak, Marianne E. Eybye, Xuling Zhu, Eleonora Guadagnin, Rebecca A. White, Lisa M. Rice, Andrea L. Frassetto, Stephen Strom, Carl Jorns, Paolo G. V. Martini, Ewa Ellis

**Affiliations:** 1 Division of Transplantation Surgery, Department of Clinical Science, Intervention and Technology (CLINTEC), Karolinska Institutet, Stockholm, Sweden; 2 PO Transplantation, Karolinska University Hospital Huddinge, Stockholm, Sweden; 3 Division of Pathology, Department of Laboratory Medicine, Karolinska Institutet, Stockholm, Sweden; 4 Moderna Inc, Cambridge, MA 02139 USA

**Keywords:** Liver diseases, Molecular medicine

## Abstract

Alpha 1-antitrypsin (AAT) deficiency arises from an inherited mutation in the SERPINA1 gene. The disease causes damage in the liver where the majority of the AAT protein is produced. Lack of functioning circulating AAT protein also causes uninhibited elastolytic activity in the lungs leading to AAT deficiency-related emphysema. The only therapy apart from liver transplantation is augmentation with human AAT protein pooled from sera, which is only reserved for patients with advanced lung disease caused by severe AAT deficiency. We tested modified mRNA encoding human AAT in primary human hepatocytes in culture, including hepatocytes from AAT deficient patients. Both expression and functional activity were investigated. Secreted AAT protein increased from 1,14 to 3,43 µg/ml in media from primary human hepatocytes following mRNA treatment as investigated by ELISA and western blot. The translated protein showed activity and protease inhibitory function as measured by elastase activity assay. Also, mRNA formulation in lipid nanoparticles was assessed for systemic delivery in both wild type mice and the NSG-PiZ transgenic mouse model of AAT deficiency. Systemic intravenous delivery of modified mRNA led to hepatic uptake and translation into a functioning protein in mice. These data support the use of systemic mRNA therapy as a potential treatment for AAT deficiency.

## Introduction

Alpha 1-antitrypsin deficiency (AATD) is an underdiagnosed autosomal recessive disease primarily affecting the lungs and the liver. Laurell and Eriksson first described the condition based on observations of electrophoresis of sera from patients in 1963 in Sweden^1^. It is often misdiagnosed as chronic obstructive lung disease. The disease is caused by an inherited mutation in the SERPINA1 gene^2^. Over 100 different genotypes have been identified but clinical disease manifestation is predominantly seen in the homozygous severe form named PiZZ^3^. The serine protease inhibitor Alpha 1-antitrypsin (AAT) is abundantly circulating in blood. It is an acute phase reactant displaying anti-inflammatory and immunomodulatory effects^4–6^. Circulating AAT mainly regulates and neutralizes the proteolytic effects of neutrophil elastase in the lungs^7^. The severe alpha 1-antitrypsin deficiency is caused by a point mutation resulting in an unfavourably charged protein leading to aberrant folding and protein aggregation. Liver pathogenesis is driven by aggregated protein accumulation in the rough endoplasmic reticulum^8^. The liver is the major producer of AAT and hepatocytes are therefore highly affected by the cytotoxic effect of protein aggregates. Misfolding and accumulation in the liver leads to low level of circulating protein and this deficiency in turn leads to pulmonary manifestation and premature development of chronic obstructive pulmonary disease.

Hepatic involvement ranges from severe neonatal cholestasis requiring liver transplantation to first manifestation of liver cirrhosis in the fifth decade of life. The majority of patients go unnoticed and up to 15% develop cirrhosis and hepatocellular carcinoma^9^.

Augmentation with purified plasma AAT is the only medical therapy currently available for AATD^10–13^. This treatment is associated with high economic cost and it is used to protect lung function in patients with affected lungs and its efficacy is debated^14^. Lung transplantation is an option in cases of severe pulmonary insufficiency and liver transplantation is employed in advanced cases of cirrhosis/HCC but due to organ shortage this advanced treatment is not available to all patients with severe deficiency.

Safe, efficient and non-viral modified messenger RNA (mRNA) delivery is emerging as a promising therapy for many diseases with several trials ongoing^15,16^. The modified mRNA is produced as a mature single stranded mRNA to be available for translation by the cellular machinery to generate bioactive proteins^17^. Unwanted immune responses are avoided by using pseudouridine incorporation, which can enable repeated administrations^18^. This platform offers unique temporal options where it is possible to get a pulse-like expression during a limited time frame. Also, modified mRNA has several advantages over conventional gene therapy approaches of virus- and DNA-based vectors, avoiding risk of tumorigenicity, suboptimal genome integration, ectopic sustained expression, and disadvantages related to development and production^19–21^. This emerging technology has therefore been suggested for application in enzyme replacement therapies and is of particular interest for inborn errors of metabolism^22^.

Given that modified mRNA has been demonstrated to be useful in several areas and *in vivo* delivery is now feasible, we explored its application targeting human hepatocytes and investigated AAT encoding modified mRNA on primary human hepatocytes.

## Results

### Patient characteristics

In total 16 human hepatocyte isolations were performed. Most of the donor tissue originated from male donors (87,5%). A variety of underlying diagnosis was used (Table 1), most common being tissue from organ donors either not suitable for clinical transplantation or from remnant tissue not used in size-reduced transplantations (37,5%). Another frequent diagnosis was resected non-tumorous tissues from colorectal cancer liver metastasis 25%. In three cases (18,8%) we were able to perform cell isolations from AATD patients undergoing liver transplantation using explanted AATD livers. All cases were verified with SNP genotyping assay, which confirmed that all three deficient cases indeed displayed the Z allele, all other cases displayed M variants, (Table 1).

**Table 1:** Summary of patient characteristics.

ID	Diagnosis	Sex	Age (year)	Cell viability %	Allele
HF370	AATD	M	68	80	Z
HF371	Organ donor	M	6	71	M
HF384	AATD	M	5	74	Z
HF395	FAP	M	31	82	M
HF399	Organ donor	F	40	84	M
HF403	Organ donor	M	68	78	M
HF414	Organ donor	M	27	82	M
HF423	Colorectal metastasis	M	73	79	M
HF424	Colorectal metastasis	M	68	67	M
HF425	Cholangiocarcinoma	M	73	71	M
HF426	Unknown tumour	M	71	80	M
HF433	Colorectal metastasis	M	73	66	M
HF435	Colorectal metastasis	F	81	66	M
HF440	Organ donor	M	63	80	M
HF455	AATD	M	0.8	78	Z
HF456	Organ donor	M	12	77	M
Diagnosis	n (total = 16)	Sex			
A1ATD	3 (18,8%)	Female	2 (12,5%)		
Cholangiocarcinoma	1 (6,3%)	Male	14 (87,5%)		
Colorectal metastasis	4 (25%)	Cell isolation (mean)			
FAP	1 (6,3%)	Age (years)	47,5 (0.8–81)		
Organ donor	6 (37,5%)	Tissue mass (g)	258,4 (41–584)		
Unknown tumour	1 (6,3%)	Cell viability (%)	75,9 (66–84)		

AATD: Alpha 1-antitrypsin deficiency,

FAP: Familial Amyloidotic Polyneuropathy,

M: male, F: female.

### Transfecting primary human hepatocytes

**Figure 1:**
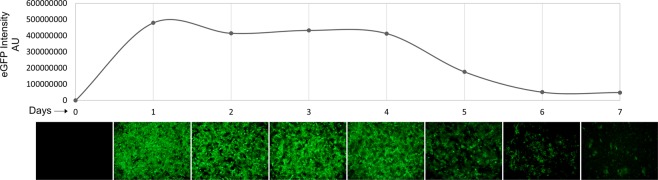
eGFP transfection time course. Primary human hepatocytes were transfected with eGFP encoding mRNA by lipofection in culture. Protein expression peaked after 1 day and continued robust expression was seen 4 days as assessed by immunofluorescence imaging. Corrected total fluorescence was measured and plotted.

Cells were allowed to attach and repolarize in culture for 24 hours prior to transfection procedure. Several conditions were tested including different mRNA concentrations, Lipofectamine™ 2000:mRNA ratios and different transfecting reagents (Fig. S1). Addition of 3 µg mRNA proved sufficient for robust transduction (Fig. S2). Less mRNA resulted in lower expression and increased concentration did not equate to significant improvement or longer expression. Transfection efficacy was evaluated by enhanced green fluorescent protein (eGFP) transduction by manual inspection and corrected total measured fluorescence. eGFP expression was observed 2 hours after addition of mRNA to hepatocytes. Maximal expression was observed after 24 hours with a subsequent tapered reduction in expression the following 6 days in culture (Fig. 1).

### Lipofection using AAT modified mRNA resulted in intracellular translation and protein expression

**Figure 2:**
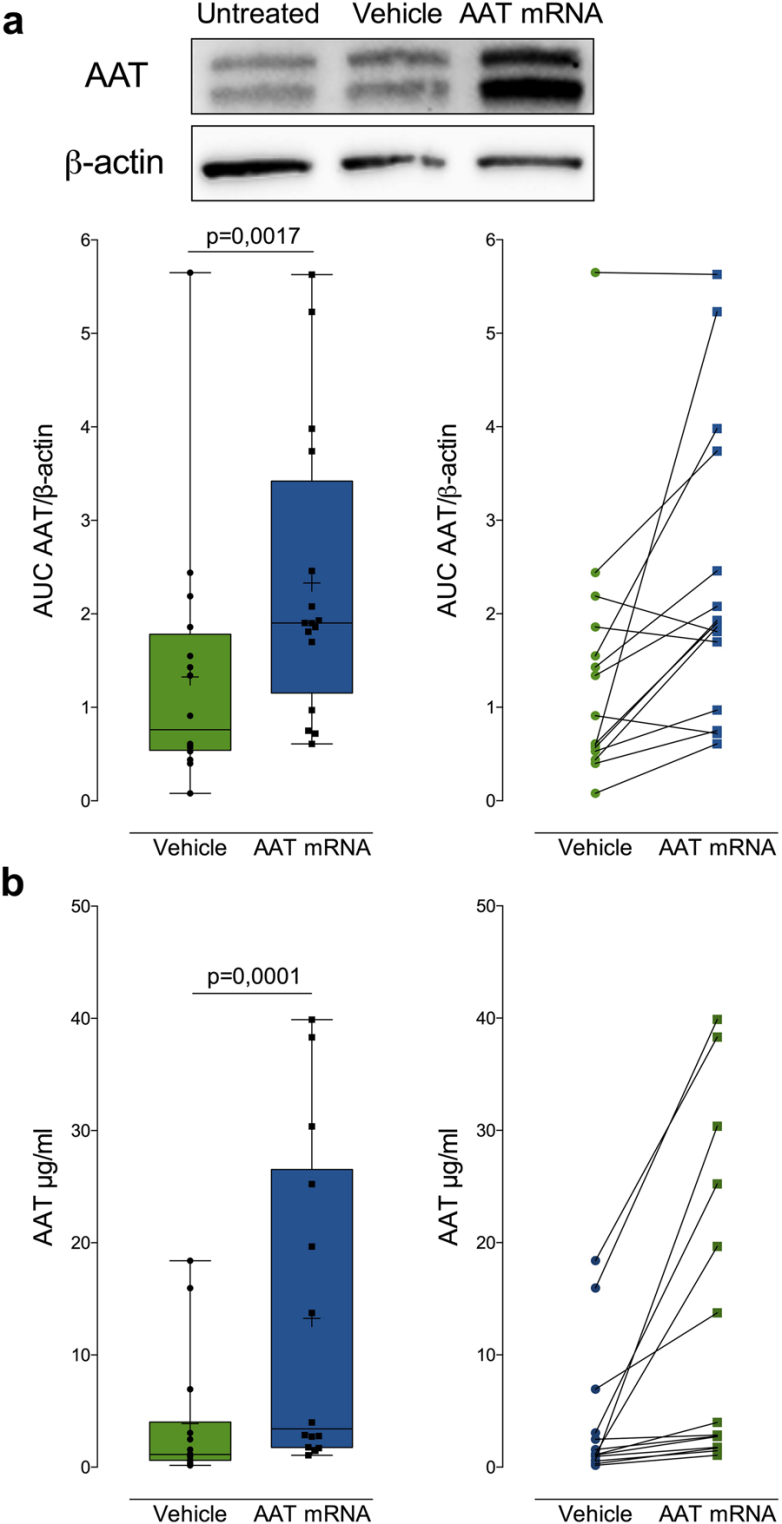
AAT encoding mRNA translation into protein by hepatocytes and secretion into the extracellular space. **(a)** An increase in AAT production was observed in hepatocytes exposed to modified mRNA. Gel separation and immunoblotting revealed increased AAT in the treated group (n = 16). **(b)** ELISA of secreted AAT protein in culture media showed an increase in total amount of secreted AAT protein (n = 14).

Modified mRNA encoding AAT was delivered by lipofection using the Lipofectamine™ 2000 reagent following an optimized protocol developed from eGFP mRNA (Fig. 1). Expression was allowed for 48 hours and AAT was accumulated both intracellularly and secreted into culture media. Intracellular AAT was examined by western blot and modified mRNA-exposed hepatocytes were compared to Lipofectamine™ 2000 liposome (vehicle) exposed hepatocytes. A significant increase in AAT protein expression could be observed (Fig. 2). All samples were analysed by normalizing AAT levels to the internal loading control β-actin in the same sample. Median relative area under curve (AUC) increased from 0.76 to 1.90 units (Wilcoxon matched-pairs signed rank test, p = 0,0017). Increased intracellular AAT protein expression level was seen in all cases but four, where one case displayed no change and three cases showed a decrease in AAT expression level.

### AAT protein is produced in hepatocytes and undergo extracellular transport

AAT is a protein circulating in blood and it is therefore secreted into circulation after expression in producing cells, mainly hepatocytes. It was therefore critical to examine if the AAT protein produced from modified mRNA could undergo post-translational modifications and subsequent extracellular secretion. Hepatocytes were cultured for 48 hours after transfection and AAT was quantified in culture media using ELISA. All cases displayed an increase in AAT production to some extent although the increase was modest in some cases (Fig. 2b). There was an overall three-fold significant increase in AAT protein secretion (median 1,14 to 3,43 µg/ml, Wilcoxon matched-pairs signed rank test, p = 0,0001).

### Modified mRNA AAT product exhibit protease inhibitor activity

**Figure 3:**
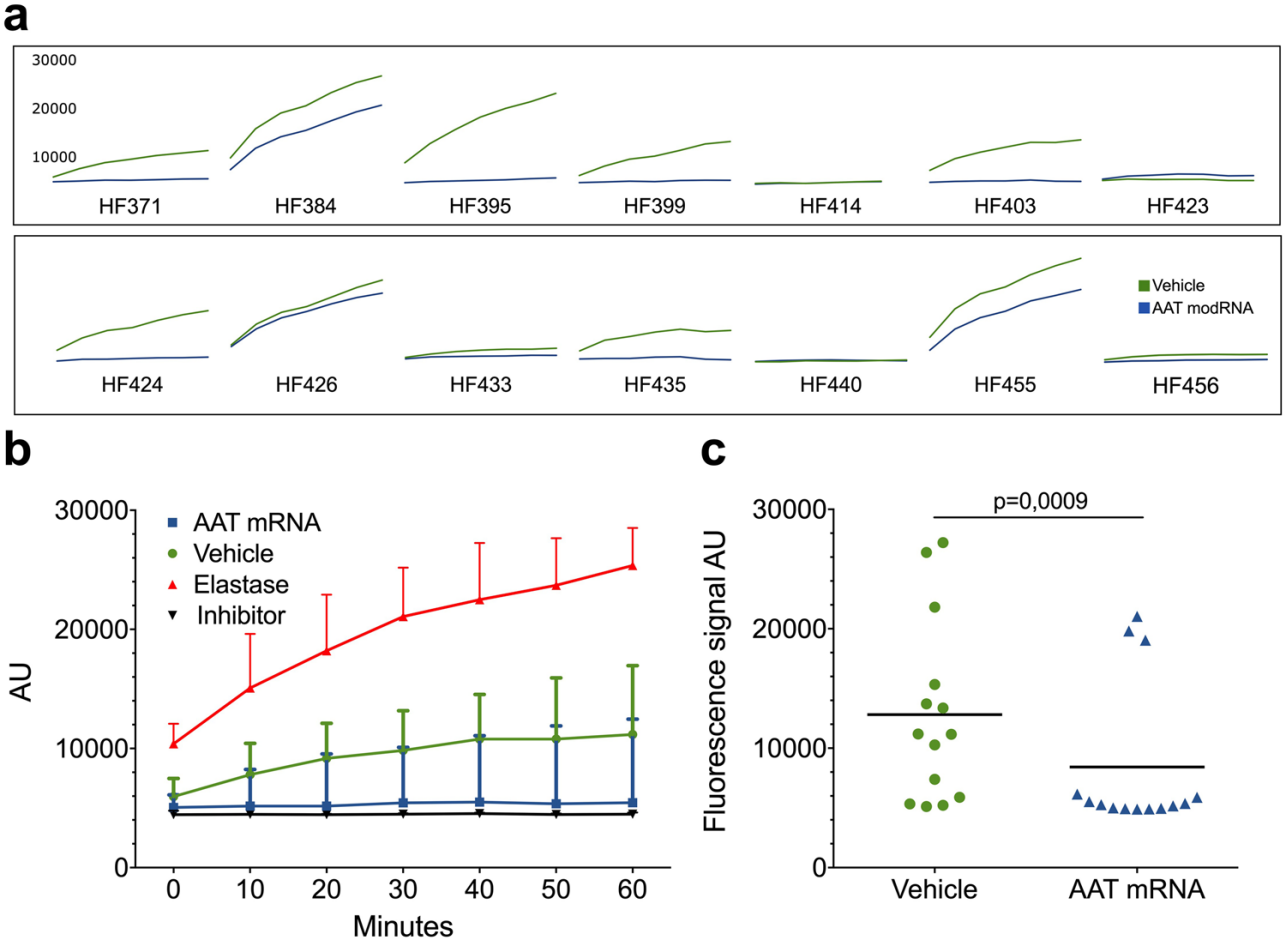
Elastase activity assay shows retained AAT protease inhibition and protein function. **(a)** Elastase activity was measured in n = 14 cases. A majority of cases display a decreased elastase activity when incubated with conditioned media from cell cultures. **(b)** Median elastase activity of all cases showed a robust inhibition of elastase activity in the presence of conditioned media displaying similar kinetics as control elastase inhibitor. **(c)** End-point (60 minutes) analysis showed significant (Wilcoxon matched-pairs signed rank test) decrease in elastase activity in conditioned media from cells treated with AAT modified mRNA.

An elastase activity assay was used to test the bioactivity of AAT produced from modified mRNA. The assay measures porcine pancreas elastase activity by digesting bovine neck ligament derived fluorescent conjugate elastin. If left uninhibited the enzyme digests elastin and the fluorescent signal is increased accordingly over time. However, in the presence of an inhibitor this reaction is haltered, and the fluorescent signal is reduced. Conditioned media from hepatocytes exposed to modified mRNA was added to a mixture of both elastase enzyme and fluorescent conjugate elastin substrate and compared to unconditioned media from the same case, e.g. hepatocytes only exposed to Lipofectamine™ 2000. A positive control, elastase inhibitor (*N-*Methoxysuccinyl-Ala-Ala-Pro-Val-chloromethyl ketone) or a negative control (reaction buffer) was also used for internal assay validation (data not shown). Time-course curve of reactions from all cases are presented in (Fig. 3a), with a median of all reactions summarized in (Fig. 3b) showing the dynamic progression of the reaction. A clear distinction was observed over time between the two groups, almost all cases displayed reduced elastase activity in conditioned media compared to control media from non-transfected cells. Also, at end-point of the reaction, which was set to 60 minutes, the difference between treated and untreated groups was evident (Fig. 3c) (p = 0,0009, Wilcoxon matched-pairs signed rank test).

### Intravenous delivery of lipid nanoparticle embedded mRNA

**Figure 4:**
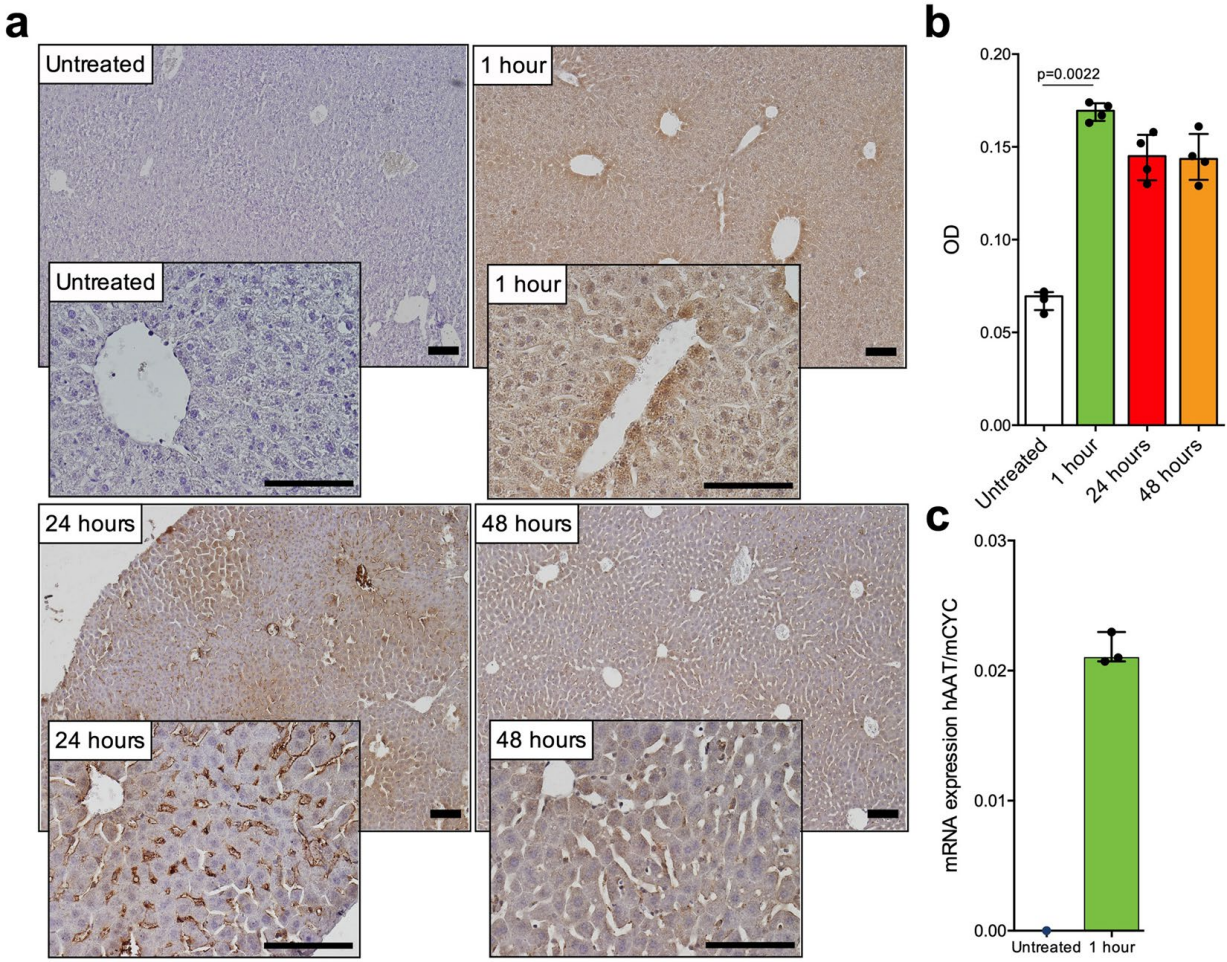
Expression of human AAT protein following intravenous injection of modified mRNA in wild type mouse. Modified mRNA in LNP formulation was administered as a one-time intravenous (tail-vein) injection at 0.5 mg/kg into C57BL/6 mice (n = 3) and liver tissue was collected 1 hour; 24 hours and 48 hours post injection. (**a)** Liver tissue was stained for human AAT protein, which revealed a robust intracellular expression in hepatocytes followed by accumulation in the sinusoidal space, scalebar 100 µm. Tissues were collected and stained at 1, 2, and 24 hours post injection and was compared to untreated control animal were no human AAT protein was expressed. (**b)** DAB staining changes were compared between time points (Kruskal-Wallis test, median with interquartile range). (**c)** RT-qPCR was used to confirm the delivery of AAT encoding mRNA into liver tissue, where AAT signal passed threshold at 27 cycles and no signal was detected in untreated samples.

Lipid nanoparticle (LNP) formulated mRNA encoding AAT was given as a single dose and delivered intravenously via a tail vein injection to wild type C57BL/6 mice (n = 3) (Fig. 4). RT-qPCR using human specific probe for AAT was used to confirm the delivery and presence of AAT encoding mRNA in mouse liver 1 hour post injection. AAT passed detection threshold at 27 cycles in mouse liver tissue from treated animals whereas no signal could be detected in the untreated sample (Fig. 4c). Immunohistochemistry was used to examine liver tissue from animals 1, 24 and 48 hours post injection. AAT protein could be detected throughout the liver parenchyma inside hepatocytes 1 hour post injection of mRNA. The expression was global however a perivenous concentration could be observed. Relative signal quantification and measurement of optical density showed significant (Kruskal-Wallis, p = 0,0022) increase of signal with rapid and strong expression (Fig. 4). Protein expression could be detected at 24 hours post injection. Although intracellular expression remained, the protein seemed to concentrate in the sinusoidal space by 24 hours, indicating extracellular secretion. Continued protein expression was still detected 48 hours after mRNA injection.

### NSG-PiZ mouse model of AATD

**Figure 5:**
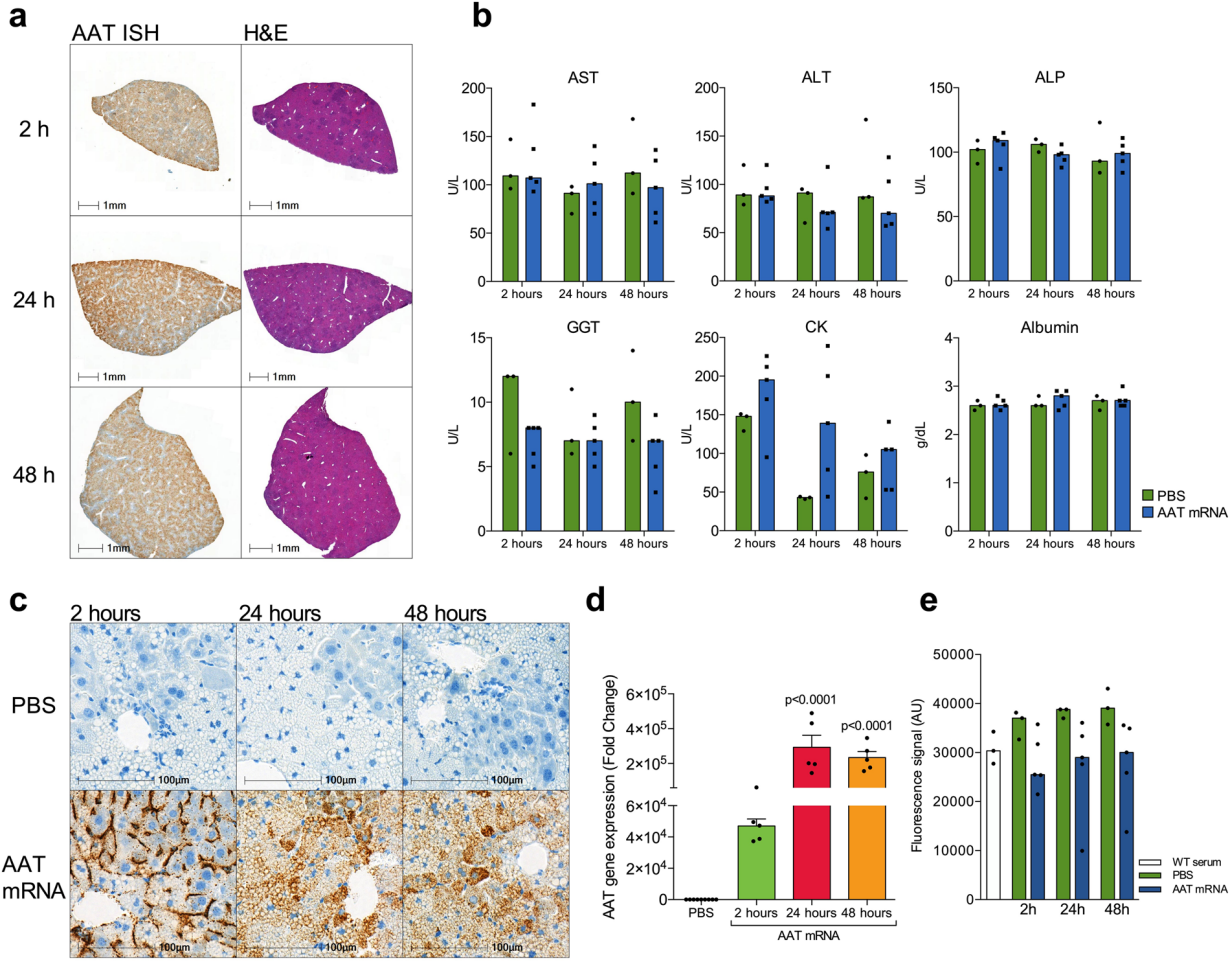
*In vivo* delivery and analysis of modified mRNA encoding human AAT into the NSG-PiZ mouse model of AATD. **(a)** Whole liver was sectioned and *in* situ hybridization was used to show global distribution of mRNA in the liver following intravenous delivery by tail-vein injection. **(b)** Biochemical analysis did not reveal any sign of cellular stress or damage caused by mRNA delivery. **(c)** Most of the mRNA can be detected by *in situ* hybridization in the liver sinusoids 2 hours after injection. The mRNA subsequently relocated into hepatocytes by 24 and 48 hours after injection. Hepatocytes with and without globules contained mRNA. **(d)** A large amount of mRNA could be detected by qPCR in analysed liver tissue. **(e)** Elastase activity assay showing inhibition in serum from animals receiving AAT mRNA and PBS treated animals.

Similar results were recapitulated in the NSG-PiZ mouse model of AATD. Fifteen male NSG-PiZ mice were treated with intravenous mRNA encoding human AAT and 9 animals with PBS control injections. In the treated group, mRNA was delivered at 1 mg per kg bodyweight and five animals per group were euthanized at 2, 24 and 48 hours after mRNA delivery. Harvested liver tissue showed high expression of human AAT from modified mRNA delivery, examined by qPCR (Fig. 5d). Robust expression could be seen at all time points. Furthermore, *in situ* hybridization showed human AAT modified mRNA delivery into liver sinusoids and subsequent intracellular delivery into hepatocytes (Fig. 5a,c). Both hepatocytes containing AAT globules and hepatocytes without globules displayed uptake of AAT modified mRNA (Fig. 5c). Serum was collected and examined for elastase inhibition. Serum from animals treated with modified mRNA displayed a trend of higher inhibitory capacity compared to PBS control animals although this did not reach statistical significance (Fig. 5e). Delivery of modified mRNA did not affect the cellular morphology of the liver (Fig. 5a,c) nor did it affect markers of hepatocyte damage as measured by aspartate aminotransferase (AST), alanine aminotransferase (ALT), alkaline phosphatase (ALP), creatine kinase (CK), gamma-glutamyl transferase (GGT), and Albumin, (Fig. 5b).

## Discussion

The only currently available treatment for AATD apart from transplantation is augmentation therapy consisting of weekly or bi-weekly intravenous delivery of exogenous plasma derived AAT protein and has pulmonary loss of function as the primary indication^12,23,24^. Augmentation therapy is not currently used for AATD related liver disease and it is not available in all countries. Manifested liver cirrhosis with or without hepatocellular carcinoma is treated with liver transplantation, however transplantation is limited by organ shortage making this therapy scarce and not available for all patients in need. Liver disease in AATD is also largely undiagnosed and frustratingly undertreated. Recombinant production of human proteins is an option, however, these proteins produced by either bacteria, plant cells or other mammalian cells may differ from endogenously produced proteins and pose complications and high production cost.

Previous important preclinical trials for modified mRNA have successfully shown the efficacy of modified mRNA in LNP-formulations in delivery and translation in murine disease models of both acute intermittent porphyria, methylmalonic academia and most recently arginase deficiency^22,25,26^. These promising results have led to the pursuit of clinical translation. An important step towards true translational realization is safe and reliable delivery of mRNA with adequate translation and a production of a fully functional protein. It has been demonstrated that the LNP-formulation used is biodegradable and a potent vehicle for delivery of mRNA. The mRNA cargo is protected in blood, both stopping mRNA degradation and reducing the risk of immunization^27,28^.

Using the same technology and platform as in previous studies we investigated the potential use of modified mRNA therapy as a treatment option for AAT-deficiency. Firstly, we demonstrate the mRNA delivery into primary human hepatocytes in culture. Secondly, we showed that the delivered mRNA is correctly translated into AAT-protein by the cellular machinery. Furthermore, post-translational modifications seem to be intact as demonstrated by final protein size on gel separation and immunoblotting (Fig. 1). Also, most importantly, the produced and secreted protein demonstrates protease inhibitory function (Fig. 3). Although no purification of AAT newly produced from delivered mRNA was performed, the overall protease inhibition was examined and displayed an increased inhibitory capacity. Increased inhibition is an effect of newly synthesized protein, closely mimicking the effect of chemical protease inhibitor (Fig. 3b).

Time course experiments using eGFP-encoding mRNA showed a prolonged protein expression in primary human hepatocytes of up to 7 days following transfection (Fig. 1). Intracellular levels of AAT protein were analysed by protein extraction and immunoblotting and revealed increased AAT protein in treated cells (Fig. 1). Not all cases showed increased intracellular AAT. Three cases were hepatocytes from AATD patients which exhibited high baseline AAT content, most likely due to accumulated and entangled intracellular AAT protein and up regulated AAT production. In contrast to intracellular analysis of AAT, the extracellular measurement showed an increase in AAT protein in all cases (Fig. 2). A more robust increase in the extracellular fraction was to be expected as mature AAT protein is normally secreted into systemic circulation. A three-fold increase in median expression was observed, mean concentration of AAT protein in cell culture media showed an increase from 3,88 to 13,26 µg/ml. Larger fold change of AAT expression using mRNA has been previously reported in immortalized cell lines or from fibroblasts and hepatocyte like cells derived from fibroblasts from AATD patients^29,30^. In our studies the fold change was lower and we believe this is because we are measuring AAT production in primary human hepatocytes that are main producers of AAT and closely recapitulate clinical settings. Larger fold-expression is to be expected when examining non-AAT producing cell types. Furthermore, our cohort included three cases of primary human hepatocytes from AAT deficient patient with the severe Z allele mutation (Table 1). We were able to observe high elastase activity in these cases when using media from untreated cells. The high elastolytic activity was however reduced in the presence of mRNA indicating production of non-mutant AAT protein with inhibitory capacity even in hepatocytes with severe Z mutation.

Delivery of mRNA is paramount in order to translate to clinical treatment. In this study we used the LNP-formulated version of the modified mRNA for *in vivo* settings. LNP mediated mRNA systemic delivery was performed by tail vein injection in wild type C57BL/6 mice and also into the transgenic NSG-PiZ mouse model of AATD. Delivered mRNA was detected using RT-qPCR in the liver as early as 1 hour and up to 48 hours after initial dosing. LNPs display high uptake in the liver as previously demonstrated^22,26^, which is advantageous as AAT is mainly produced in hepatocytes. In NSG-PiZ mice, the delivered mRNA was detected by both RT-qPCR and RNA *in situ* hybridization of AAT in liver tissue. Uptake was global throughout the liver and importantly, both hepatocytes containing globules and those without globules showed robust uptake of modified RNA (Fig. 5a). This indicates that cells affected by protein accumulation and globule formation can receive mRNA encoding the normal protein variant and may produce the healthy and functioning AAT protein variant, which may in turn lead to recovery from aggregation induced cell damage. Ameliorating effects, especially on globule size and distribution will require longer observational time, which was not in the scope of this study. Respiratory improvement and pulmonary recovery may be seen earlier than potential liver recovery, this is however not possible to assess in animal models as the NSG-PiZ model does not display pulmonary manifestations of AATD. Delivery of mRNA showed no increase in hepatocyte damage or death as indicated by analysis of liver associated biomarkers in blood (Fig. 5b) and lack of morphological changes in liver parenchyma (Fig. 5a).

Translation of modified mRNA was observed in wild type mice, where AAT protein could be detected 1-hour post injection. Initially, a zonation could be observed where perivenous hepatocytes were expressing higher levels of AAT 1-hour post injection (Fig. 4a). The expression pattern changed as time elapsed to reveal an enrichment of AAT protein in the sinusoidal space. We speculate that this is a result of AAT protein being secreted from hepatocytes and or by uptake of liver sinusoidal endothelial cells. These results are in line with previous extensive *in vivo* characterization of hepatic delivery of LNP and mRNA translation in mice^22,25,26^. Serum from NSG-PiZ mice receiving modified mRNA displayed the same increased protease inhibitory capacity that was observed in primary human hepatocytes as compared to untreated controls. Increased protease inhibition further shows the translation and secretion of functioning AAT protein *in vivo* and in a relevant animal model for AATD.

Another way of delivery that has been explored is administration of mRNA in the lungs. Potentially this could provide a local translation and functional levels of AAT. However, hepatic delivery and production closely mimics the endogenous AAT production and function. It is also arguably more beneficial in regards to liver disease causing mutant AAT accumulation in the liver. We showed in a previous study that SERPINA1 gene expression could be down-regulated in human hepatocytes following the addition of purified exogenous AAT protein. Even though we were unable to specifically examine the mutant protein due to methodological limitations, we argue that restoring adequate levels of AAT protein might relieve liver toxicity by reducing the amount of mutant protein that is produced and entangled in hepatocyte of deficient individuals^31^. Restoring adequate circulating levels of AAT is indeed not only important for lung function but may also be beneficial for reduction of insult to hepatocytes caused by protein accumulation through a possible negative feed-back regulation. Modified mRNA-based replacement therapy may also extend treatment to patients with non-severe mutations such as S or heterozygous Z alleles for whom pulmonary deficiency may not be present although these mutations may cause severe liver disease^32^. Other therapeutic approaches are underway and some are showing promising results, where bone marrow derived stem cell transplantations have shown partial recovery of liver damage in the PiZ mouse model of AATD^33^.

In conclusion, we have demonstrated a way to deliver mRNA encoding human AAT into primary human hepatocytes where mRNA is translated into functioning protein. Also, LNP formulated mRNA was delivered *in vivo* in mice and was successfully translated in hepatocytes and secreted to systemic circulation. LNP formulations of other mRNA candidates are currently being tested in phase 1 trials, which could enable clinical translation of the modified mRNA technology also for AAT replacement therapy.

## Methods

### Ethical considerations

The study was approved by the Swedish Ethical Review Authority. Written informed consent was obtained from all patients for participation in the study. All tissue from donor livers had consent for use in clinical transplantation and for use in research. All animal experiments were conducted in accordance with both European Union, national and local (Karolinska Institutet) guidelines and with approval from The Swedish Board of Agriculture. Animal subjects were kept to minimum numbers sufficient to convey scientific conclusions in each experiment, in accordance with the principles of The Three Rs.

### Patient tissue specimen for hepatocyte isolation and culture

Liver tissue used for hepatocyte isolation was acquired from resected tissue from patients undergoing liver resection surgery following primary/secondary tumours or extirpated livers from AATD patients being liver transplanted at our centre. Written informed consent was obtained from all patients for participation in the study. Also, liver tissue was obtained from donor livers rejected for clinical transplantation. Isolations were conducted with approval from the Swedish Ethical Review Authority. The isolation process followed a previously established three-step collagenase perfusion technique^34,35^. Cell recovery and viability were determined using trypan blue exclusion method. Cells were cultured in William’s E medium (Sigma-Aldrich, Stockholm, Sweden) supplemented with 20 mM 4-(2-hydro- xyethyl)-1-piperazineethanesulfonic acid (HEPES), 2 mM glutamine, 12 nM insulin, 100 nM dexamethasone, 50 nM amphotericin B and 0.01 M gentamicin. Cells were plated in 12 well plates (Corning Life Sciences, Tewksbury, MA) at a density of 1×10^6^ cells per well. The hepatocytes were cultured under standard culture conditions in 37 °C, 5% CO_2_ and in a humidified atmosphere.

### Genotyping and qPCR

Single Nucleotide Polymorphism (SNP) genotyping was used on all included human samples. Allelic discrimination method was performed using TaqMan® assays on an ABI Step-One Plus (Applied Biosystems, Foster City, CA) real-time PCR instrument according to manufacturer’s instruction. Pre-designed SNP Genotyping Assay probes were purchased from Applied Biosystems. PiS allele, rs1758 (assay ID: C_594695_20); PiZ allele, rs28929474 (assay ID: C_34508510_10); PiM2/M4 allele, rs709932 (assay ID: C_2895146_20) PiNull allele, rs28929473 (assay ID: C_63321235_20). Total RNA was extracted from mouse liver tissue with the TRIzol® reagent (Invitrogen, Carlsbad, CA) and was reverse-transcribed using the high-capacity cDNA reverse transcription kit (Applied Biosystems, Thermo Fisher Scientific, Carlsbad, CA) into cDNA according to manufacturer’s instructions. Specific TaqMan® assays specific for housekeeping gene mouse Cyclophilin A (assay ID Mm02342429_g1) and human AAT gene SERPINA1 (assay ID Hs01097800_m1) containing primers and hydrolysis probes. Quantitative real-time PCR was performed on an ABI Step-One Plus instrument.

For NSG-PiZ animals, 100 mg of flash frozen liver tissue was homogenized and total RNA isolation was performed using RNeasy Mini Kit (Qiagen, MD) according to manufacturer’s recommendations. First strand cDNA was synthesized from total RNA (2 µg) using qScript XLT cDNA SuperMix (Quantabio, MA). Real-time qPCR was performed using Power SYBR Green (Life Technologies, MA) and ran on CFX384 Touch Real-Time PCR detection System (Bio-Rad, CA). Primers used in our experiments were from IDT (MA). The relative amount of all mRNAs was calculated using comparative CT method (ΔΔCt). HPRT was used as internal control.

### mRNA production and formulation

Human AAT mRNA modifications were achieved using established approaches^36^. Both eGFP and human AAT mRNA was synthesized *in vitro* and formulated with lipid nanoparticle (LNP) as previously described^22^. Plasmid encoding the T7 RNA polymerase promotor followed by 5′ untranslated region (UTR), open reading frame (ORF), 3′UTR, and polyA tail was overexpressed in *E. coli*, linearized, and purified to homogeneity. The mRNAs were synthesized by T7 RNA polymerase transcription, where uridine triphosphate was substituted with 5-methoxy uridine triphosphate. Cap 1 was utilized to improve translation efficiency. After purification, the mRNA was buffer exchanged into sodium citrate buffer and stored at −20 °C until use.

LNP formulations were prepared using a modified procedure of a method previously described^37^. Formulation of mRNA was performed through ethanol injection nanoprecipitation by mixing acidified RNA and lipids dissolved in ethanol at a 3:1 ratio (aqueous:ethanol) at a lipid molar ratio of 50:10:38.5:1.5 (ionizable: fusogenic: structural: PEG). After pH adjustment, the mRNA-loaded lipid nanoparticles were buffer exchanged into PBS solution and stored at 4 °C until use. Final particle size and encapsulation were <100 nm and >80%, respectively, with endotoxin below 10 EU/mL.

### Transfection

Delivery of modified mRNA was optimized for use in cultured primary human hepatocytes. Lipofectamine™ 2000, 3000, RNAiMax (Thermo Fisher Scientific, Inc., MA) and TransIT® -mRNA transfection kit (Mirus BIO, LLC, WI) were tested for efficiency and Lipofectamine™ 2000 transfection reagents proved best suited. Modified mRNA was allowed to thaw and mixed with Opti-MEM® I (Thermo Fisher Scientific) reduced serum media. Lipofectamine™ 2000 was also diluted in Opti-MEM® I and incubated at room temperature for 5 minutes. Diluted Lipofectamine™ 2000 and modified mRNA were mixed at a ratio of 3:1, where 9 µl Lipofectamine™ 2000 reagent was used for 3 µg modified mRNA and allowed for lipoplex formation for 15 minutes at room temperature. Lipoplexes were added and mixed into culture media of cultured hepatocytes. Lipofection using enhanced green fluorescent protein (eGFP) encoding modified mRNA was used as control of transfection. Empty liposomes without mRNA content acted as vehicle control, this was selected as minor cytotoxic effect was observed using eGFP as control. Cellular cytotoxicity of GFP has been previously reported^38,39^.

### Protein extraction and immune blotting

Cultured hepatocytes were disrupted and lysed following 48 hours incubation with modified mRNA. Culture media was removed and cells were washed in cold phosphate-buffered saline (PBS). Cells were lysed with radioimmunoprecipitation assay (RIPA) buffer (R0278, Sigma-Aldrich, Stockholm) supplemented with protease inhibitor cOmplete**™**, Mini, EDTA-free Protease Inhibitor Cocktail (04693159001, Roche Diagnostics GmbH, Mannheim, Germany), centrifuged and stored in −80° for further analysis. Protein concentrations were determined by BCA assay following manufactures instructions (5000002, Bio-Rad Laboratories AB, Stockholm, Sweden). Lysates (20 µg protein/lane) were separated by SDS-PAGE (Any kD™ Mini-PROTEAN^®^ TGX™ Precast Protein Gels, 4569033, Bio-Rad Laboratories AB, Stockholm, Sweden) and used for immunoblotting. Membranes were incubated with anti-SERPINA1 (AAT) (1:2000 dilution, HPA001292, Atlas Antibodies AB, Stockholm, Sweden) and anti-β-actin (1:2000 dilution, ab8227, Abcam plc, Cambridge, UK) antibodies. Membranes were developed using WesternBright ECL Kit (K-12045-D20, Advansta Inc, San José, CA) and imaged with Vilber Lourmat UV-instrument (Vilber Lourmat, Collégien, France). Blots were analysed and AAT signal was normalized to β-actin signal using ImageJ software (National Institutes of Health, Bethesda, MD)^40^.

### AAT Enzyme-linked immunosorbent assay

AAT protein concentration in cultured cell media was determined by enzyme-linked immunosorbent assay (ELISA). Culture media was collected 48 hours after transfection analysed for AAT content by Human alpha 1 Antitrypsin ELISA Kit (SERPINA1) (ab108799, Abcam plc, Cambridge, UK) according to the manufacturer’s instructions.

### AAT activity assay

AAT function and protease inhibition were determined by EnzCheck Elastase Assay Kit (E-12056, Molecular Probes, Eugene, OR) according to manufacturer’s instructions. Briefly, soluble bovine neck ligament elastin has been labelled with a fluorescent conjugate that is quenched. The substrate is digested in the presence of elastase or other proteases to yield a fluorescent signal. Working solutions of substrate and provided elastase enzyme from pig pancreas were prepared and combined with cell culture media from vehicle or mRNA treated cells or from serum samples from NSG-PiZ mice. Negative and positive controls were used. Fluorescence intensity was measured in a dynamic fashion using the CLARIOstar microplate reader (BMG LABTECH GmbH, Ortenberg, Germany) with readings every 10 minutes for a total of 60 minutes.

### *In vivo* delivery

Experiments, animal housing, handling, and experimental procedures/protocols were approved and performed in accordance with guidelines and regulations of The Swedish Board of Agriculture and local guidelines by Karolinska Institutet. C57BL/6 male mice were used in this study. Animals were kept in a temperature-controlled environment with a 12-h/12-h light- dark cycle, with a standard chow and water ad libitum. Mice were injected with human AAT modified mRNA at 0,5 mg/kg body weight in LNP-formulation as provided by Moderna Inc (Cambridge, MA) via the tail vein. Animals were euthanized 1, 24 and 48 hours post injection and liver tissue was both snap-frozen and fixed in 10% formalin for immunohistochemistry. NSG-PiZ animal experiments were conducted at Moderna Inc facilities in accordance with local regulations and recommendations. Male NOD.Cg-*Prkdc*^*scid*^ *Il2rg*^*tm1Wjl*^ Tg(SERPINA1*E342K)#Slcw/SzJ, (NSG-PiZ) mice, acquired from The Jackson Laboratories (Bar Harbor, ME) were divided into two groups where 15 animals receiving intravenous mRNA encoding human AAT and 3 animals receiving PBS control injections. Modified mRNA was delivered at 1 mg per kg bodyweight and animals were euthanized at 2, 24 and 48 hours after mRNA delivery. Liver tissue was stored both by snap freezing in liquid nitrogen and saved in −70 °C and by 10% formalin fixation. Blood, lung and intestines were also collected.

Liver associated and general cell damage blood biomarkers aspartate aminotransferase (AST), alanine aminotransferase (ALT), alkaline phosphatase (ALP), creatine kinase (CK), gamma-glutamyl transferase (GGT), and Albumin were analysed in blood samples from all NSG-PiZ animals by IDEXX BioAnalytics (North Grafton, MA).

### Immunohistochemistry

Liver tissue was fixed in 4% buffered formalin solution, dehydrated and paraffin embedded. Embedded tissue was sectioned into 5 µm thick sections and further rehydrated. Sections were pressure cooked for 30 minutes in buffered citrate antigen retrieval solution pH = 6 prior to incubation with Background Buster blocking solution (NB306-50, Innovex Biosciences Inc., Richmond, CA) for 20 minutes. Primary rabbit anti-human SERPINA1 antibody (1:200) (HPA001292, Atlas Antibodies) was used and sections were incubated one-hour at room temperature. ImmPRESS® HRP Anti-Rabbit IgG (Peroxidase) Polymer Detection Kit (MP-7451, Vector Laboratories, INC, Burlingame, CA) reagent was used to stain and visualize according to manufacturer’s instructions. DAB staining was compared by measuring reciprocal DAB intensity using ImageJ software according to methods described by Fuhrich *et al*.^41^.

### mRNA *In Situ* Hybridization

mRNA *in situ* hybridization was performed using RNAscope technology on the automated Leica BOND RX autostainer platform using the RNAScope® 2.5 LS Reagent Kit-BROWN from Advanced Cell Diagnostics (Newark, CA). The following RNAscope probes were used in this study. An exclusive target probe with proprietary sequences was designed by ACD to target human SERPINA1 mRNA and avoiding cross-reactivity to mouse or rat SERPINA1. Control probes to the housekeeping gene Mus musculus peptidylprolyl isomerase B (Ppib), mRNA (cat no 313918, ACD) was used as a positive control or the bacterial gene dihydrodipicolinate reductase (DapB) (cat no 312038, ACD) as a negative control was also used as a quality control check for tissues. Slides were processed using a Leica staining protocol per the ACD user manual Document Number 322100-USM (ACD). All images were captured at 20X magnification with the Pannoramic 250 Flash III (3DHISTECH, Budapest, Hungary) digital slide scanner.

### Statistical analysis

Data is presented as median with interquartile range where non-parametric data is expressed. Individual values are reported, and boxplots or bars show median (line) mean (+ sign) and interquartile values and range are displayed. Two-tailed p values <0.05 were considered significant. Non-parametric Wilcoxon matched-pairs signed rank test was applied for comparisons between two groups, and Kruskal-Wallis test for comparisons between more than two groups, as normal distribution could not be assumed. Statistical analysis was performed in Prism version 6 (GraphPad Software Inc., San Diego, CA).

## Supplementary information

Supplementary information.
