# Supplementary material for: Systemic modified messenger RNA for replacement therapy in alpha 1-antitrypsin deficiency
Source: Sci Rep. 2020 Apr 27;10:7052. doi: 10.1038/s41598-020-64017-0 (PMC7184591; doi:10.1038/s41598-020-64017-0)
Supplement: Supplementary file 1 — Supplementary information. [file 41598_2020_64017_MOESM1_ESM.docx]

**Systemic modified messenger RNA for replacement therapy in alpha 1-antitrypsin deficiency**

**Supplementary**

Ahmad Karadagi^1,2^, Alex G. Cavedon^4^, Helen Zemack^1^, Greg Nowak^2^, Marianne E. Eybye^4^, Xuling Zhu^4^, Eleonora Guadagnin^4^, Rebecca A. White^4^, Lisa M. Rice^4^, Andrea L. Frassetto^4^ Stephen Strom^3^, Carl Jorns^2^, Paolo G. V. Martini^4^, Ewa Ellis^1*^

**Affiliations**

^1^Division of Transplantation Surgery, Department of Clinical Science, Intervention and Technology (CLINTEC), Karolinska Institutet, Stockholm, Sweden

^2^PO Transplantation, Karolinska University Hospital Huddinge, Stockholm, Sweden

^3^Division of Pathology, Department of Laboratory Medicine, Karolinska Institutet, Stockholm, Sweden

^4^Moderna Inc, Cambridge, MA 02139, USA

***Corresponding author:**

**Ewa Ellis, Ph.D.**

Liver Cell Lab F67, Division of Transplantation Surgery, CLINTEC, Karolinska University Hospital Huddinge, SE-141 86 Stockholm, Sweden;

Tel: +46 8 585 800 86

email: [ewa.ellis@ki.se](mailto:ewa.ellis@ki.se)


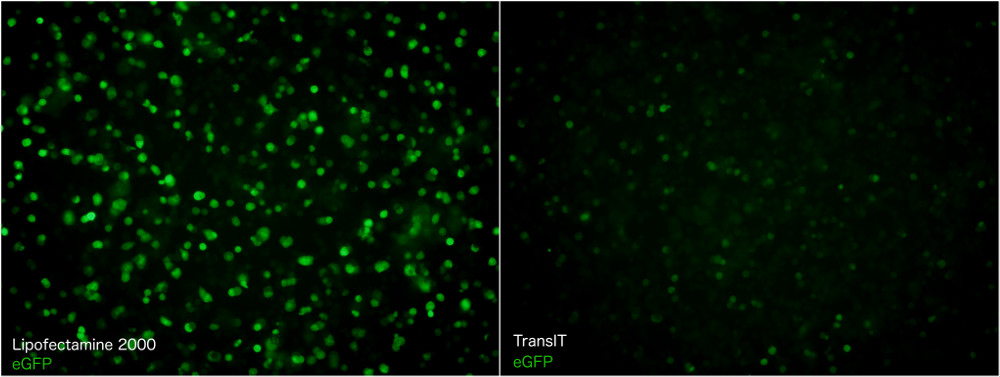


**Figure S1.** **Comparing lipofecting agents.** Transfection using lipoplexes formed by both Lipofectamine™ 2000 (Thermo Fisher Scientific, Inc., MA) and TransIT® -mRNA transfection kit (Mirus BIO, LLC, WI) lipofection agents using mRNA expressing eGFP was performed. Lipofectamine 2000 proved higher efficiency as detected by visual inspection.


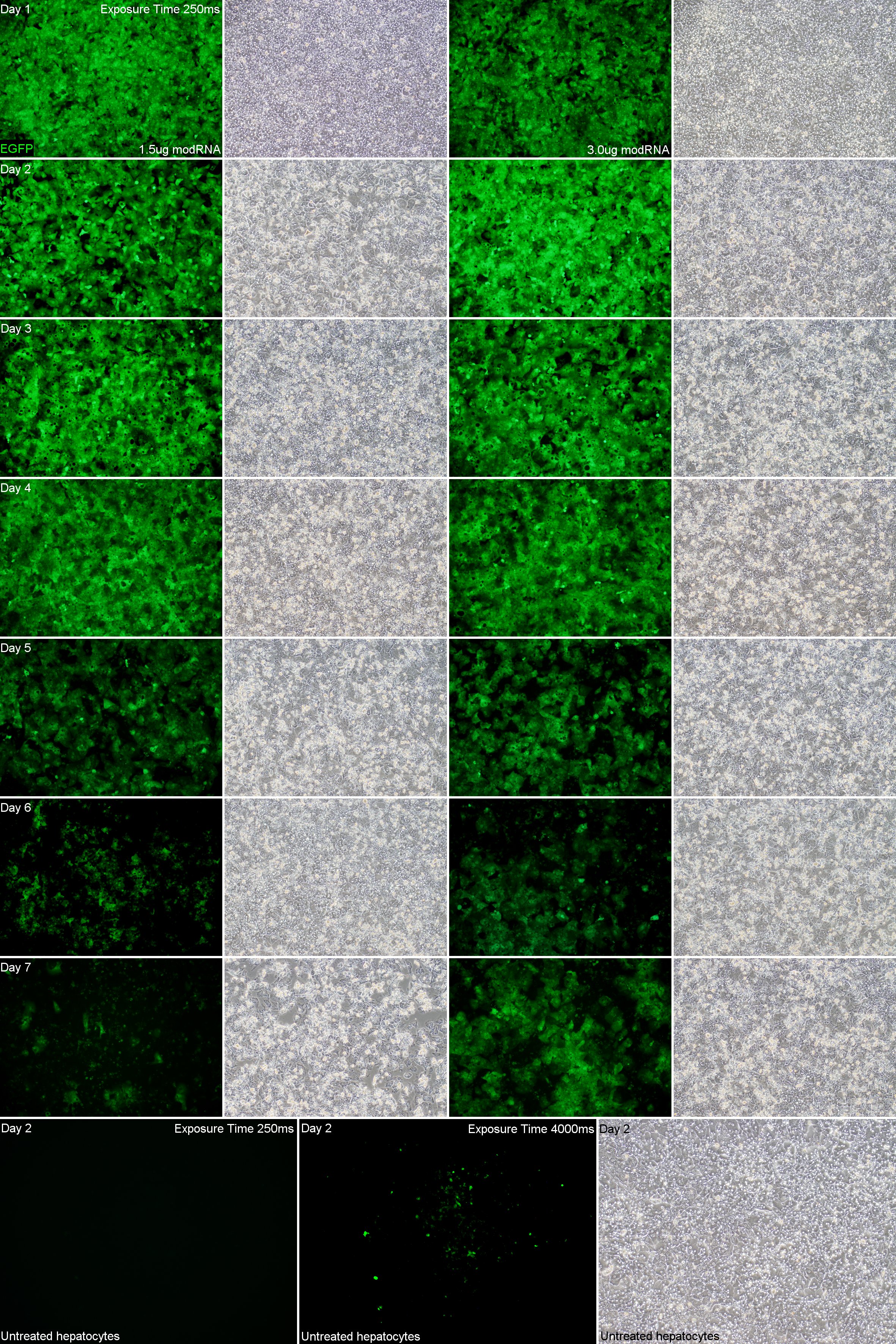


**Figure S2.** **Time-course, eGFP expression.** Primary human hepatocytes were transfected with mRNA encoding eGFP using Lipofectamine™ 2000. Robust expression was seen the first 4 days with subsequent decrease. Both 1,5 µg and 3 Both 1,5µg and 3µg mRNA per 1x10^6^ cells translated into high protein expression, 3µg was however more efficient and was selected for further analysis. Even higher 6µg concentration was examined without showing further improvements, data not shown.

**
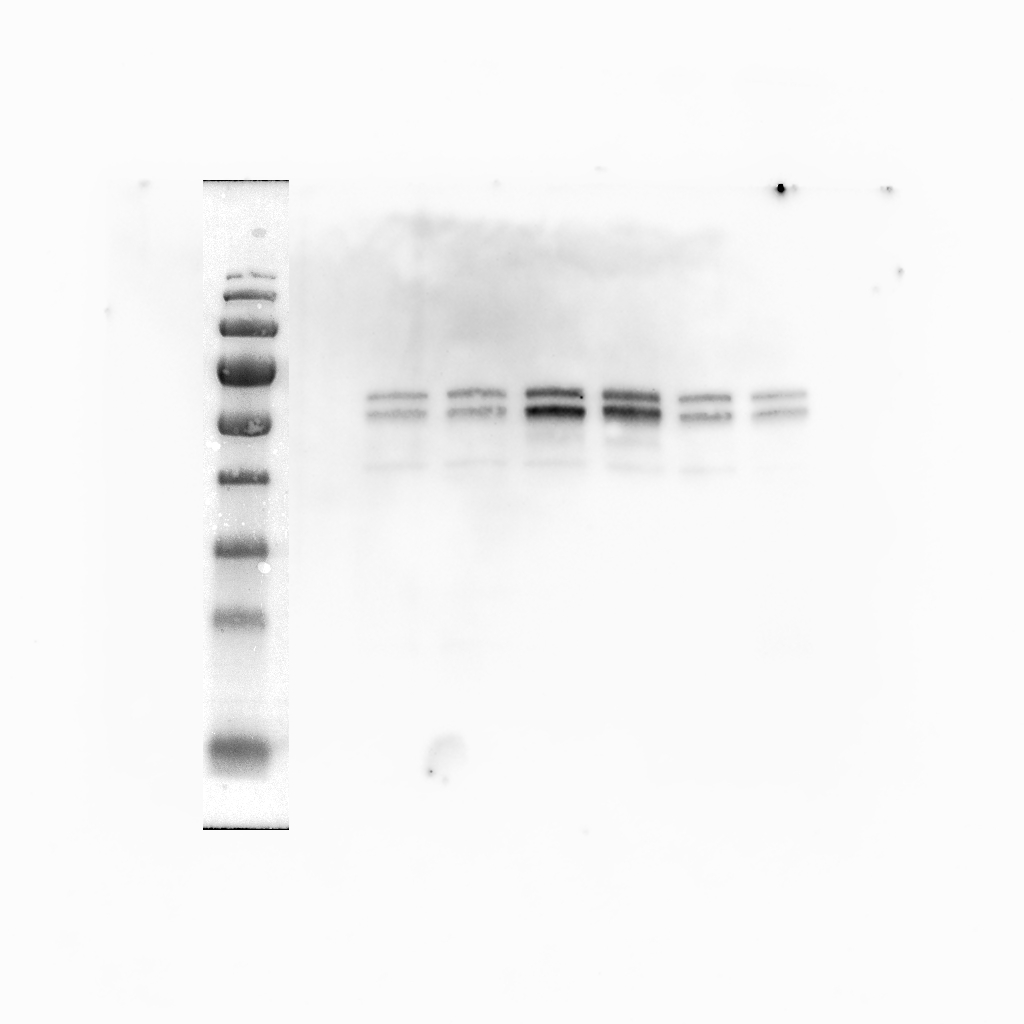

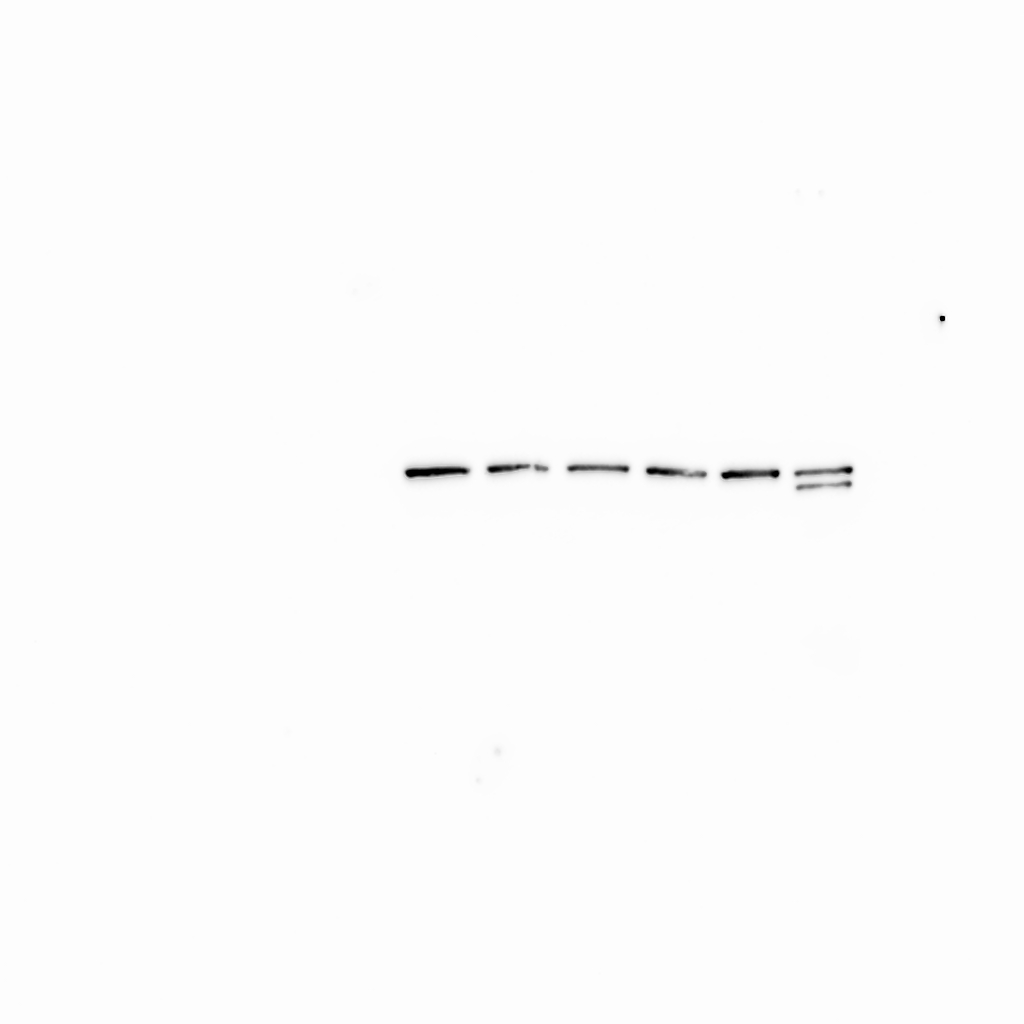
**

**Figure S3. Full-size western-blot gels.** Full-size photo of representative gels used in **Figure 2a.** Gels were loaded with 20µg protein from lysates from: untreated cells; vehicle; AAT mRNA; AAT-Flag mRNA; LNP formulated AAT mRNA and LNP formulated AAT-Flag mRNA. **a** Rabbit anti-human SERPINA1 (1:2000 dilution, HPA001292, Atlas Antibodies AB, Stockholm, Sweden) **b** Rabbit anti human Beta Actin (1:2000 dilution, ab8227, Abcam plc, Cambridge, UK)
